# Gender differences in discharge dispositions of emergency department visits involving drug misuse and abuse—2004-2011

**DOI:** 10.1186/s13011-017-0114-5

**Published:** 2017-05-30

**Authors:** Jennifer I. Manuel, Jane Lee

**Affiliations:** University of Washington School of Social Work, Seattle, WA USA

**Keywords:** Emergency department, Substance abuse, Discharge disposition, Gender

## Abstract

**Background:**

Drug use-related visits to the emergency department (ED) can undermine discharge planning and lead to recurrent use of acute services. Yet, little is known about where patients go post discharge. We explored trends in discharge dispositions of drug-involved ED visits, with a focus on gender differences.

**Methods:**

We extracted data from the 2004–2011 Drug Abuse Warning Network, a national probability sample of drug-related visits to hospital EDs in the U.S. We computed weighted multinomial logistic regression models to estimate discharge dispositions over time and to examine associations between gender and the relative risk of discharge dispositions, controlling for patient characteristics.

**Results:**

The final pooled sample included approximately 1.2 million ED visits between 2004 and 2011. Men accounted for more than half (57.6%) of all ED visits involving drug misuse and abuse. Compared with women, men had a greater relative risk of being released to the police/jail, being referred to outpatient detox or other treatment, and leaving against medical advice than being discharged home. The relative risk of being referred to outpatient detox/drug treatment than discharged home increased over time for men versus women.

**Conclusions:**

Greater understanding of gender-based factors involved in substance-related ED visits and treatment needs may inform discharge planning and preventive interventions.

## Background

During the past 10 years, ED visits involving drug use have increased significantly, especially among specific drug categories and sub-populations [1–3]. For example, between 2004 and 2008, the rate of prescription opioid-related visits to the ED increased by 111% [4], partially attributed to the increased likelihood of ED visits among women [5]. Although a greater percentage of men die from drug overdose, the rate of deaths involving drug overdose among women has significantly increased since 1999 [1].

Drug-involved ED patients are at high risk for co-occurring health and mental health problems, unstable unemployment, and legal trouble [6–11]. They are also more socio-economically disadvantaged and uninsured and therefore more likely to use the ED as their primary source of care instead of substance use or primary care services [12–14]. While EDs are required to stabilize patients, stabilization alone is not sufficient to return patients to their baseline level of functioning. Successful recovery often requires substance use treatment that extends beyond acute hospitalization. The ED as a point of clinical contact serves as an important setting to screen and identify patients with drug use disorders who may benefit from brief interventions in the hospital and referrals to treatment following discharge [15, 16].

Improving the continuity of care is important with regard to substance use as the majority of people who need treatment for substance use disorders do not obtain treatment [17, 18]. Women, in particular, are underrepresented in substance use treatment programs, and more likely than men to face barriers to accessing treatment [19–21]. Correspondingly, the extant literature highlights gender disparities in health and social outcomes of individuals with substance use disorders, with women experiencing greater health and mental health comorbidity and financial difficulties [19, 21, 22]. While the influence of gender on patterns of substance use and treatment entry is widely acknowledged [19, 23], less is understood regarding the pathways by which men and women enter substance use treatment. Several studies have indicated that men are more likely to enter treatment via the criminal justice system, while women are most often referred from community and other health providers [20].

The ED is an important clinical entry point for patients with drug use disorders, with increasing attention on the ED as an opportune site to intervene and refer to substance use treatment [8]. Yet, little is known regarding the range of care to which patients are referred post discharge and how gender may play a role in the process [8]. While one study found that women presenting to the ED are less likely than men to receive referrals to outpatient detox treatment [3], less is known about gender differences in other discharge dispositions. Several studies have shown that a significant number of patients with drug use disorders leave the hospital or ED against medical advice (AMA) [24–26]. Both substance use and male gender have been independently associated with an increased likelihood of leaving AMA [27–30]. Yet, there are few studies to demonstrate how gender is implicated in discharge AMA [31], discharges to police, home discharges, or other discharges among patients with drug use disorders. Recently, through a consensus process, experts in emergency medicine have called for more research on gender differences in substance use in the ED setting [32].

As the ED may serve as a potential link between patients with drug use disorders and substance use treatment [33], data from the ED may elucidate information on gender differences in patterns of substance use and access to services for the most vulnerable drug using population [34]. Despite progress towards developing effective gender-specific programming, such as services that address issues around pregnancy, child care, and trauma [35], recent evidence suggests that programs specifically designed for women in substance use treatment may be declining [36]. At the same time, the Affordable Care Act (ACA) of 2010 included a number of reforms aimed at integrating health and behavioral health services and improving access to and the quality of health care services, especially for patients with substance use disorders [37]. For example, new health insurance plans offered through the marketplace are required to cover treatment for substance use disorders that is equal to coverage for medical and surgical treatment, expanding the benefits of the Mental Health Parity and Addiction Equity Act of 2008. In addition, the evidence-supported Screening, Brief Intervention, and Referral to Treatment (SBIRT) for risky alcohol use is a covered benefit in many states [37] and may address important access barriers for women.

Given the need to better understand how individuals enter substance use treatment, we explored discharge dispositions in a nationally representative sample of drug-involved ED visits from 2004 to 2011. Our main objectives were to describe characteristics of drug-involved ED visits and examine gender differences in discharge dispositions and explore whether these differences changed over time. We developed hypotheses that were based on our assessment of existing literature and trends in drug use and drug use outcomes among men and women. Specifically, we hypothesized that discharge disposition differs as a function of gender, with a greater risk of home discharges, general hospital admissions, and transfers to another facility, and a lower risk of adverse- and detox-related (inpatient and outpatient) discharges among women compared to men.

## Methods

We analyzed data from the Drug Abuse Warning Network (DAWN) from 2004 to 2011, the final year of data collection. Conducted by the Substance Abuse and Mental Health Services Administration (SAMHSA), DAWN was a network of more than 250 hospitals in the United States that produced a nationally representative data system of drug-related visits to hospital Emergency Departments (EDs). Between 2004 and 2011, DAWN relied on a probability sample of hospitals that met the following eligibility criteria: non-Federal; short stay; general surgical and medical; located in the United States; and has a 24-h ED. Hospitals were selected using stratified simple random sampling with oversampling in select metropolitan areas. Post-stratified weights were applied to the data from sampled hospitals to generate results representative of the target population.

**Fig. 1:**
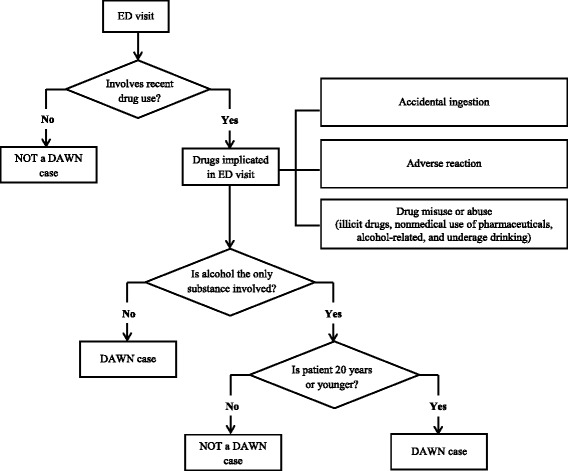
Determination of a DAWN case

Data were collected retrospectively via annual review of medical records for patients treated in the ED. A trained DAWN reporter in each member facility reviewed medical records to identify ED visits related to drug use. Specifically, the DAWN reporter assessed three key areas of a patient’s ED chart (i.e., patient’s chief complaint, clinicians’ assessment, and/or diagnosis generated from the *International Classification of Diseases*) and examined eligibility criteria to determine if the ED visit was for a condition induced by or related to drug use. A drug was defined by DAWN as any substance that is “(a) used as a medication or in the preparation of medication; (b) an illicit substance that causes addiction, habituation, or a marked change in consciousness; (c) or both” [38]. Up to 22 drugs could be reported for each ED visit, and could include both intentional and accidental use of drugs. Hence, an ED visit related to drug use for therapeutic purposes as prescribed by a doctor was also captured as a DAWN case. Figure 1 illustrates the determination of a DAWN case.

**Fig. 2:**
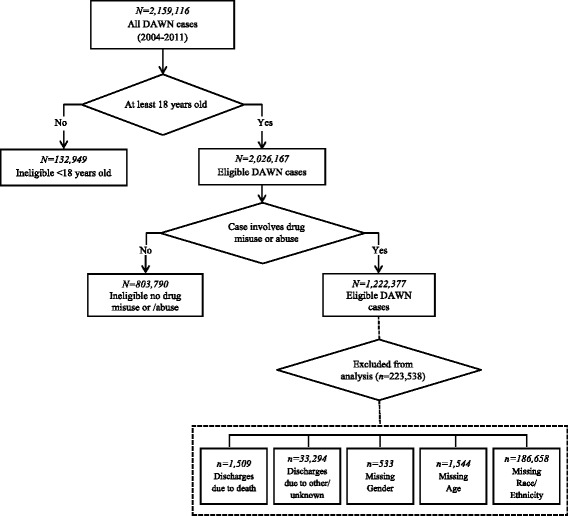
Derivation of the DAWN analytical sample, 2004–2011

For the current study, we identified ED visits related to misuse and abuse of illicit and prescription drugs of patients aged 18 years or older. Figure 2 illustrates a flow chart of DAWN cases included in the study (*N* = 1,222,377). DAWN defined drug misuse and abuse as ED visits that included illicit drugs, nonmedical use of pharmaceuticals, alcohol-related visits, and underage drinking [38]. Illicit drug categories included cocaine, heroin, marijuana, synthetic cannabinoids, amphetamines, methamphetamine, ecstasy, gamma-hydroxybutyric acid, flunitrazepam (Rohypnol), ketamine, lysergic acid diethylamide (LSD), phencyclidine (PCP), hallucinogens, or nonpharmaceutical inhalants (e.g., sniffing model airplane glue). Nonmedical use of pharmaceuticals included patients who took a higher than prescribed dose of medication, patients who took a drug prescribed for another person, patients who were maliciously poisoned by another person, and patients with documented substance abuse involving prescription drugs. ED visits involving alcohol were documented if alcohol was used in combination with other drugs for patients 21 years and older. It is important to note, however, that these alcohol-related visits included visits that involved drug misuse or abuse for which no illicit drug use or use of nonmedical prescription drugs were recorded in the case report. Most of these cases involved patients who were documented as taking other pharmaceuticals as prescribed and attempted suicide, were seeking detox, or had adverse drug reactions. For patients under 21 years, alcohol was documented alone and in combination with other drugs. Mutually exclusive drug misuse and abuse categories were created for this analysis: alcohol only; nonmedical use of prescription drugs only; illicit drugs only; illicit drugs with alcohol; nonmedical use of prescription drugs with alcohol; illicit drugs with nonmedical use of prescription drugs; and illicit drugs with nonmedical use of prescription drugs and alcohol.

The primary outcome variable was ED discharge disposition, which indicates where the patient went after leaving the ED. Discharge disposition included seven mutually exclusive categories, which were reflected in the medical records: discharged home, referred to outpatient detox/drug treatment, inpatient detox or psychiatric hospital admission, general hospital admission, transferred to another facility, released to the police/jail, and left against medical advice. We excluded drug-involved visits for discharge dispositions related to death given the relatively small number of observations (*n* = 1509), as well as dispositions due to other reasons, including not specified and unknown (*n* = 33,294).

DAWN collected limited data to characterize ED visits, which included gender (male and female), age (18–20, 21–34, 35–54, and 55 years or older), and race/ethnicity (non-Hispanic white, non-Hispanic Black or African American, Hispanic, and other). We excluded missing data on gender (*n* = 533) and age (*n* = 1544), which comprised less than 1% of the sample, and race/ethnicity (*n* = 186,658), which comprised about 15% of the sample. No missing data were reported for age or any of the alcohol/drug categories. Based on comparisons between included and excluded observations, a greater percentage of excluded patients were younger, Black, and used cocaine (*p* < .01), although the percentage differences were less than 2%.

### Statistical analysis

All statistical analyses were performed using Stata/MP, version 14.0 and accounted for sampling design effects and sampling weights. Bivariate and multivariate tests of significance were examined using design-based logistic regression and multinomial logistic regression, respectively. Variances were computed using Taylor series approximations, which is Stata’s default variance estimation method. We used standard suppression rules for annual ED visit estimates if they reached a threshold defined by a relative standard error greater than 50% or an unweighted count less than 30 [38]. Analyses used survey weights to produce nationally representative estimates of drug-involved ED visits.

We calculated weighted proportions of demographic variables, illicit and prescription drug categories, and alcohol use by the total sample and by gender. We used logistic regression to compare these proportions between women and men. We used unadjusted logistic regression models to examine within-gender changes in discharge dispositions over the study period. The regression models were stratified by gender and included calendar year as a linear term to assess potential trend changes in dispositions over time from 2004 to 2011 for women and men. We used GraphPad Prism 7 to graph time trends of discharge dispositions by gender.

For the main analysis, we computed weighted multinomial logistic regression (MLR) models to examine the independent effects of variables on the likelihood of a patient’s discharge disposition per the categories described above. All models controlled for categorical predictor variables, including gender, age, race/ethnicity, and alcohol, illicit, and prescription drug categories. We used a linear term for calendar year in the final multivariate models. To assess the potential for non-linear time effects, we modeled time dependence using quadratic, cubic*,* and categorical terms for calendar year. However, the results were virtually unchanged. As such, we opted for more parsimonious models by including time as a continuous predictor in our models.

We conducted our analyses in a series of steps. First, we examined the main effects of gender on discharge disposition. Next, we included two-way interaction terms for gender by year to examine changes in discharge disposition over time between men and women. No significant gender-by-time effects were found, and thus we report findings from the main effects model. Finally, we conducted multinomial logistic regression models of discharge dispositions separately by gender to explore gender-specific effects. Results from the gender-stratified models were consistent with the full model and are available upon request.

The MLR framework depends on the assumption of independence of irrelevant alternatives (IIA) [39]. Under this assumption, the relative distribution of the discharge disposition categories will be virtually unaffected by the addition or removal of any additional category. Using the Hausman test for survey data, this assumption was not violated. Model fit statistics for survey data are presented with the MLR estimates in the results section. We used an alpha value of *p* < .01 to reduce the risk of type I error due to multiple testing and the large sample size. This study was approved by the institutional review board at New York University as exempt from oversight under the category of studying existing and publicly available data.

## Results

### Sample characteristics

Table 1 shows the weighted percentages and unadjusted logistic regression models of characteristics of ED admission visits comparing men versus women. Of the 14.2 million ED visits, approximately 42.4% were women and 57.6% were men. Results from the unadjusted logistic regression models reveal that, compared to women, men were less likely to be aged 55 years or older and white. ED visits by men were less likely to involve prescription drugs only (with or without alcohol), and more likely to involve illicit drugs only (with or without alcohol), as compared to women. Men compared to women were less likely to be discharged home and to have a general hospital admission, and more likely to be released to the police/jail, referred for outpatient detox/drug treatment, admitted to inpatient detox or psychiatric hospital care, and leave against medical advice.

**Table 1:** Characteristics of ED Visits Involving Drug Misuse or Abuse, DAWN 2004–2011

	Total (*N* = 14,245,776)	Men (*n* = 8,203,524; 57.6%)	Women (*n* = 6,042,252; 42.4%)	Men vs. Women^a^		
	Weighted %	Weighted %	Weighted %	Unadjusted OR	95% CI	*p*
Age (years)						
18–20	12.0	12.3	11.5	1.08	1.01–1.15	0.022
21–34	34.6	35.2	33.8	1.06	1.02–1.10	0.005
35–54	42.1	42.3	41.9	1.02	0.98–1.05	0.318
55 or older	11.4	10.3	12.8	0.78	0.74–0.82	<.001
Race/Ethnicity						
Non-Hispanic White	63.0	59.3	68.2	0.68	0.63–0.73	<.001
Non-Hispanic Black	24.0	25.6	21.7	1.24	1.14–1.35	<.001
Hispanic	11.6	13.8	8.5	1.71	1.59–1.84	<.001
Other	1.4	1.3	1.6	0.87	0.77–0.97	0.016
Drug Misuse or Abuse Category						
Alcohol only	8.7	8.6	8.9	0.97	0.89–1.05	0.433
Prescription Drugs only	30.8	23.8	40.3	0.46	0.44–0.49	<.001
Illicit Drugs only	30.4	34.2	25.2	1.54	1.48–1.61	<.001
Illicit Drugs w/ Alcohol	14.2	17.8	9.4	2.10	1.97–2.24	<.001
Prescription Drugs w/ Alcohol	6.3	5.7	7.1	0.78	0.73–0.84	<.001
Illicit Drugs w/ Prescription Drugs	6.9	6.9	6.9	0.99	0.93–1.06	0.805
Illicit Drugs w/ Prescription Drugs & Alcohol	2.7	3.0	2.2	1.34	1.23–1.47	<.001
Discharge Disposition						
Discharged Home	51.7	50.4	53.4	0.89	0.84–0.93	<.001
Released to Police/Jail	3.3	4.3	2.0	2.25	2.03–2.49	<.001
Referral to Outpatient Detox/Drug Treatment	5.1	5.5	4.4	1.27	1.15–1.42	<.001
Inpatient Detox/Psychiatric Hospital Admission	9.0	9.7	8.2	1.2	1.07–1.35	0.002
General Hospital Admission	20.1	19.1	21.5	0.86	0.81–0.91	<.001
Transferred to Another Facility	8.8	8.8	8.7	1.01	0.92–1.10	0.847
Left Against Medical Advice	2.1	2.3	1.8	1.25	1.12–1.38	<.001

Notes: The table reports weighted frequencies and percentages

^a^Unadjusted logistic regression models of sample characteristics and discharge dispositions as a function of gender. Odds ratio (OR) estimates were tested using design-based *t-*statistics with 1433 degrees of freedom

### Rates of discharge dispositions

**Fig. 3:**
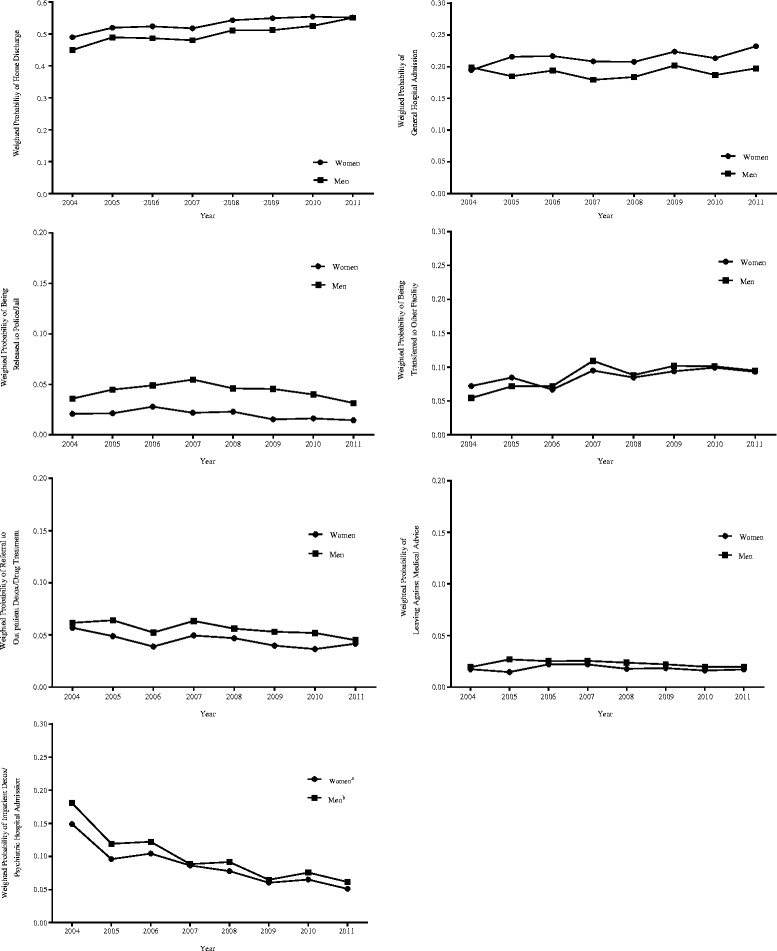
Trends in discharge dispositions from drug-involved ED visits, DAWN 2004–2011 (*N* = 14,245,776). Notes: Unadjusted logistic regression models of discharge dispositions as a function of time (continuous year) were stratified by gender. Odds ratio estimates were tested using t statistics with 1,433 degrees of freedom. ^a^Significant within-group changes over time for women (*p* < .01), ^b^Significant within-group changes over time for men (*p* < .01)

Figure 3 shows the weighted probabilities of discharge dispositions for men and women over the study period. There were limited significant within-gender changes in discharge dispositions over time. Results from the logistic regressions suggest that the largest declines were found in rates of inpatient detox or psychiatric hospital admission for both women (OR = 0.86, *t* = −3.10, design-based df = 1422, *p* < .01) and men (OR = 0.85, *t* = −2.77, design-based df = 1422, *p* < .01).

### Multinomial regression estimates of discharge dispositions

#### Released to police/jail

Table 2 shows the association of gender and other covariates with discharge disposition in a multinomial logistic regression model. Compared to women, men were associated with a greater risk of being released to the police/jail rather than discharged home. Other important risk factors of being released to the police/jail relative to home discharge were Hispanic (compared to White) race/ethnicity and visits involving illicit drugs (combined with and without alcohol, prescription drugs, or both prescription drugs and alcohol) versus alcohol only. Relative to home discharge, a lower risk of being released to police/jail was found among visits involving prescription drugs only compared to alcohol.

**Table 2:** Multinomial Logistic Regression Models of Discharge Dispositions for ED Visits Involving Drug Misuse and Abuse, DAWN 2004–2011 (*N* = 14,245,776)

	Released to Police/Jail^a^			Referral to Outpatient Detox/Drug Treatment^a^			Inpatient Detox or Psychiatric Admission^a^			General Hospital Admission^a^			Transferred to Another Facility^a^			Left Against Medical Advice^a^		
	RRR	95% CI	*p*	RRR	95% CI	*p*	RRR	95% CI	*p*	RRR	95% CI	*p*	RRR	95% CI	*p*	RRR	95% CI	*p*
Year	0.95	0.89–1.01	0.076	0.96	0.88–1.04	0.287	0.86	0.78–0.95	0.002	0.98	0.93–1.02	0.304	1.04	0.97–1.11	0.328	0.96	0.93–.99	0.034
Male Gender	1.96	1.75–2.19	<.001	1.08	0.99–1.18	0.083	1.02	0.92–1.13	0.661	1.00	0.94–1.06	0.913	1.04	0.95–1.14	0.420	1.30	1.17–1.45	<.001
Age (years)																		
18–20 (reference)	1.00			1.00			1.00			1.00			1.00			1.00		
21–34	1.18	1.02–1.37	0.024	1.46	1.27–1.68	<.001	1.91	1.66–2.19	<.001	1.86	1.61–2.15	<.001	1.60	1.42–1.80	<.001	1.60	1.35–1.89	<.001
35–54	1.21	0.96–1.51	0.100	1.36	1.17–1.59	<.001	2.26	1.97–2.60	<.001	3.13	2.60–3.76	<.001	1.82	1.62–2.05	<.001	1.81	1.50–2.18	<.001
55+	0.81	0.56–1.17	0.259	0.65	0.54–0.78	<.001	1.32	1.10–1.58	0.003	4.38	3.60–5.32	<.001	1.09	0.95–1.24	0.231	1.37	1.06–1.77	0.015
Race/Ethnicity																		
Non-Hispanic White (reference)	1.00			1.00			1.00			1.00			1.00			1.00		
Non-Hispanic Black	1.18	1.02–1.37	0.024	0.72	0.54–0.97	0.030	1.10	0.89–1.37	0.386	1.12	0.94–1.34	0.197	0.82	0.61–1.11	0.200	0.79	0.69–0.91	0.001
Hispanic	1.69	1.39–2.06	<.001	0.71	0.56–0.90	0.005	1.11	0.87–1.42	0.406	0.71	0.56–0.90	0.005	0.83	0.58–1.19	0.303	0.68	0.58–0.81	<.001
Other	1.50	0.97–2.30	0.067	0.97	0.64–1.46	0.879	1.87	1.24–2.81	0.003	1.04	0.85–1.28	0.712	1.51	1.04–2.19	0.031	0.77	0.50–1.17	0.223
Misuse or Abuse Drug Category^b^																		
Alcohol only (reference)	1.00			1.00			1.00			1.00			1.00			1.00		
Prescription Drugs only	0.70	0.56–0.88	0.002	0.48	0.40–0.59	<.001	0.63	0.48–0.83	0.001	0.76	0.67–0.87	<.001	0.73	0.55–0.95	0.020	1.34	1.02–1.77	0.038
Illicit Drugs only	1.87	1.42–2.48	<.001	1.81	1.44–2.27	<.001	1.60	1.19–2.15	0.002	0.75	0.63–0.88	0.001	0.94	0.81–1.09	0.391	2.05	1.57–2.68	<.001
Illicit Drugs w/ Alcohol	1.34	1.09–1.64	0.005	2.35	1.87–2.96	<.001	2.40	1.96–2.94	<.001	0.64	0.55–0.74	<.001	1.15	0.95–1.38	0.146	1.45	1.09–1.95	0.012
Prescription Drugs w/ Alcohol	1.09	0.80–1.48	0.597	0.95	0.74–1.22	0.677	1.10	0.85–1.42	0.479	1.02	0.88–1.17	0.832	1.13	0.94–1.35	0.188	1.42	1.01–1.99	0.041
Illicit w/ Prescription Drugs	1.7	1.33–2.17	<.001	1.29	1.05–1.59	0.018	0.87	0.67–1.13	0.301	0.99	0.84–1.17	0.938	0.98	0.82–1.18	0.855	1.76	1.29–2.39	<.001
Illicit w/ Prescription Drugs & Alcohol	2.05	1.46–2.87	<.001	1.57	1.21–2.05	0.001	1.26	0.92–1.71	0.149	0.89	0.73–1.08	0.230	1.34	1.10–1.63	0.004	1.79	1.19–2.68	0.005

^a^Compared to the probability of being discharged home (base outcome)

^b^Prescription drug use refers to nomedical use of pharmaceuticals

Relative Risk Ratio (RRR) estimates were tested using t statistics with 1331 degrees of freedom

Design-based F-statistics: F(84, 1331) = 94.89 (*p* < 0.001)

#### Referral to outpatient detox/drug treatment

No significant gender differences were found in discharges resulting in referral to outpatient detox/drug treatment compared to being discharged home. Hispanic (versus White) patients had a lower risk of being referred to outpatient detox/drug treatment relative to being discharged home. Patients who used illicit drugs (with and without alcohol) and patients who used illicit drugs with prescription drugs and alcohol (versus alcohol only) had a greater relative risk of being referred to outpatient detox/drug treatment relative to being discharged home. Visits involving only prescription drugs (versus alcohol only) had a lower risk of outpatient detox/drug treatment compared to home discharge.

#### Inpatient detox or psychiatric hospital admission

Gender did not vary significantly with respect to inpatient detox or psychiatric hospital admission. A significant time effect was found, suggesting that patients had a lower risk of being admitted to inpatient detox or psychiatric hospital care over time relative to being discharged home. Compared to alcohol only, visits involving illicit drugs (with or without alcohol) conferred a greater risk of being admitted to inpatient detox or psychiatric hospital care, while visits involving only prescription drugs were associated with a lower risk of this discharge disposition compared to home discharge.

#### General hospital admission

Relative to being discharged home, discharges resulting in general hospital admission did not vary significantly by gender. A lower risk of general hospital admission was found among Hispanic (versus White) patients, as well as visits involving prescription drugs only and illicit drugs (with and without alcohol) compared to alcohol only.

#### Transferred to another facility

No significant gender differences were found in being transferred to another facility versus home discharge. A greater relative risk of being transferred to another facility relative to being discharged home was found among visits involving illicit drugs combined with prescription drugs and alcohol versus alcohol only.

#### Left against medical advice

Men compared to women were associated with a greater risk of leaving against medical advice relative to being discharged home. A greater relative risk of leaving against medical advice compared to being discharged home was also found in visits involving illicit drugs (with and without prescription drugs) and illicit drugs combined with prescription drugs and alcohol compared to alcohol only. Relative to being discharged home, a lower risk of leaving against medical advice was found among Black and Hispanic (versus White) patients.

## Discussion

The current study helps address the recent call for more research on gender differences in drug-involved presentations to acute care settings [32]. While findings from this study highlight the gender heterogeneity in illicit and prescription drug misuse [32, 40, 41], they also point to gender differences in the discharge outcomes of patients. These differences may reflect both individual level factors that warrant differential treatment needs and structural factors that impact the ED screening, treatment and discharge process.

The most common disposition outcome was being discharged home, which is consistent with extant research that documents comparable rates of being discharged home, ranging from 54% to 88%, depending on the sample population [25, 42, 43]. Confirming part of our hypothesis, we found that men were less likely to be discharged home than women. Although the data provide limited understanding of how decisions about discharge dispositions are made, one possible explanation is that women face more barriers to accessing substance use treatment and may be less willing to accept offers for continuing care due to greater family or child care obligations, fear of social service involvement, or higher levels of financial strain compared to men [35]. Further work is needed to understand relevant and influential gender-based factors in the ED setting, as well as discharge disposition decision-making processes from the perspectives of patients and healthcare providers.

These differences may also reflect a higher likelihood of adverse discharges among men compared to women, which is consistent with our hypothesis and prior research. Specifically, the current study found a large and significant effect of men leaving against medical advice [27] and being released to the police/jail [44, 45] compared to women. Men are more likely to use illicit drugs [5], which makes them more vulnerable to arrest or incarceration. Research suggests that patients may leave against medical advice to avoid arrest or incarceration or due to fear of substance use relapse [27]. Our findings also suggest that Black and Hispanic patients are at greater risk of being released to the police/jail relative to their White counterparts. These results are consistent with reports of disproportionate rates of racial/ethnic minority men who are incarcerated for drug-involved offenses [46–48]. Additionally, Hispanic patients’ lower relative risk of being referred to outpatient detox/drug treatment corresponds to extant research that point to cultural barriers to drug treatment service utilization among Hispanic drug users [46]. Language barriers among patients and staff and/or the limited availability of bilingual outpatient treatment services may contribute to differences in referrals [47]. Greater understanding of patients’ cultural context can improve EDs’ responses to diverse populations of individuals with drug use disorders.

In contrast to our hypotheses, our study found no significant gender differences in the relative risk of being hospitalized for general, inpatient detox or psychiatric care reasons; transferred to another facility; or referred to outpatient detox/drug treatment. Discharge dispositions related to hospitalization or transfer to another facility likely involve patients with co-occurring health and/or mental health conditions or serious health complications that occurred while in the ED. We were unable to account for these factors in our analysis since the DAWN database did not include indicators of health or mental health conditions. Future research might improve our current understanding of discharge dispositions by collecting data on co-occurring problems and levels of severity, which likely influence discharge decisions.

Although our bivariate results suggest a greater risk of outpatient detox/drug treatment referrals among men compared to women, these differences disappeared in the multivariate model. In contrast to these findings, a recent study found that men were more likely to receive treatment referrals than women [3]. However, the current study controlled for type of drug, which may influence discharge disposition outcomes, whereas the former study did not. A post-hoc analysis without controlling for drug type (but controlling for gender, year, age, and race/ethnicity) found similar results as Ryoo and Choo, suggesting that differences between women and men may be largely accounted for by drug type. Nevertheless, the role of gender in shaping discharge outcomes, regardless of drug type, may point to gendered nuances in the referral process and/or substance use treatments available. Further research should examine whether the subtle effects of gender on treatment referrals warrant attention in the development of detox/drug treatment programs.

An important finding worth noting is that the rate of outpatient detox/drug treatment referrals among patients with drug use problems was low for both genders, with 5.5% in men and 4.4% in women, which is concerning. This finding is consistent with recent research that suggests a treatment referral rate of 7.3% among healthcare providers for regular/chronic drinkers [48]. Research suggests that nearly a third of ED patients have perceived unmet treatment needs for substance use problems [9]. The low rate of referrals may reflect patient motivation or other individual factors, such as lack of readiness to seek treatment or feelings of shame or stigma related to substance use. Other barriers may include system or treatment level factors, such as limited availability or accessibility of treatment programs due to cost, insurance coverage, or lack of training or resources for screening and referral in healthcare settings like the ED.

These findings reinforce the importance of national efforts to expand access to substance use treatment, including screening and brief interventions for substance and alcohol use in medical settings such as EDs. While evidence supporting the efficacy of SBIRT for addressing drug use is mixed [49, 50], gender-related factors remain important considerations for screening, intervention, and referrals, particularly for ED settings. We know from prior research that, compared to men, women are less likely to access substance use services and have greater unmet treatment needs [45]. Other research has found that women are more likely to have co-occurring mental health problems and trauma histories, which lead to poorer drug treatment response and increased risk of psychosocial problems. Although men report using more substance use services, they are more likely to be treated in ED settings and use inpatient medical services [34]. These findings are consistent with our results, which found a greater prevalence of ED visits involving drug misuse and abuse among men compared to women who visited the ED. Men who present to the ED may have different needs, such as legal involvement [34, 44], which may contribute to adverse discharge outcomes. Increased awareness of these gender-specific needs of patients with drug use disorders among ED providers may lead to greater referrals and follow-through with substance use aftercare services following ED discharge.

Implicated in a gender-specific approach to ED discharge is that providers involved in the ED discharge process take into account the male and female differences in patterns of illicit and prescription drug use, risk and protective factors for drug use, risks and benefits obtained from specific treatments, and reasons for dropping out of treatment. Hence, coordination of care with other providers and services to address, for example, mental health and child care needs among women, is critical to responding to potential barriers to accessing substance use services. The establishment of the National Institute on Drug Abuse’s Women and Sex/Gender Differences Research Program highlighted the need to integrate sex and gender effects into all aspects of drug abuse research. Correspondingly, as evidence points to gender effects in the discharge dispositions of patients with drug use disorders, ED providers have an opportunity to further tailor services and care coordination to potentially improve patient outcomes.

The DAWN dataset had both limitations and strengths. DAWN is unique because it was one of a few nationally representative samples of EDs in the United States, and unlike other national datasets it focused exclusively on drug-involved visits. A limitation of this dataset, however, involves the age of the data. This study used DAWN data collection between 2004 and 2011, the last year of data collection, and may not be representative of the current patient population. However, at present, few national databases provide comprehensive data on drug-involved ED visits with a wide range of discharge dispositions.

A further limitation is that ED medical records varied in detail and specificity, which can impact the reliability and accuracy of findings. Notably, DAWN data relied on assessments made by ED medical staff who determined which drugs were related to ED visits. Clinical biases and error among medical staff may contribute to over- or under-representation of drug misuse and misclassification of discharge outcomes. In addition, the limited demographic and clinical data provided in DAWN prevent evaluation of potential confounders. We do not have information on patients’ severity of illness, health insurance coverage, or co-occurring psychiatric or medical issues. In addition, the data on race/ethnicity are flawed as this information is often poorly documented in ED records or not released due to privacy concerns. In the current study, there was a high percentage of missing race/ethnicity data (about 15%). However, analyses were conducted with and without the race/ethnicity variable and yielded similar results. Nevertheless, the authors recognize that missing data and other excluded observations from our analyses may result in biased estimates, although the percentage differences between the included and excluded were relatively small.

## Conclusions

Assessment and identification of ED patients who are at risk for drug use disorders are important to incorporate as part of the stabilization treatment in ED settings. Our findings, based on 8 years of data from a nationally representative sample of ED visits in the United States, highlight the importance of considering the role of gender during assessment and referral of ED patients with or at risk for drug use disorders. In the current period of health care reform, our findings call attention to the need for coverage of substance use treatment and implementation of preventive interventions and discharge planning services in ED settings that can respond to the treatment needs of men and women. Our results also provide important markers to assess the changing landscape of our health care system and underscore the need for continued monitoring through similar successor surveillance systems as DAWN. Future studies are warranted to ensure access and effectiveness of these preventive interventions targeting ED patients involved in prescription or illicit drug misuse.

## Abbreviations

ACA: Affordable care act
AMA: Against medical advice
DAWN: Drug abuse warning network
ED: Emergency department(s)
SAMHSA: Substance abuse and mental health services administration
SBIRT: Screening, brief intervention, and referral to treatment
